# The role of attentional control in moderating attentional bias towards game-related stimuli in individuals with a high tendency for internet gaming addiction

**DOI:** 10.3389/fpsyt.2024.1355204

**Published:** 2024-08-27

**Authors:** Yea-Ji Heo, Gi-Eun Lee, Jang-Han Lee

**Affiliations:** Department of Psychology, Chung-Ang University, Seoul, Republic of Korea

**Keywords:** internet game addiction tendency, attentional control, attentional bias, attentional engagement bias, attentional disengagement bias

## Abstract

**Background:**

The aim of this study was to elucidate individual difference factors that modulate the attentional processing of game stimuli to explain the heterogeneity of extant findings. The current study examined whether individual differences in components of attentional control (AC-shifting and AC-focusing) moderated the link between internet gaming addiction symptom and attentional engagement to and disengagement biases from game-relevant cues.

**Methods:**

A total of 75 male undergraduate students who have played League of Legend (LOL) for more than 2 years completed well-established self-report questionnaires of internet gaming disorder symptoms and attentional control. The attentional bias toward game stimuli was measured for attentional engagement and disengagement using the attentional response to the distal vs. proximal emotional information (ARDPEI) task.

**Results:**

The results revealed that attentional control was a significant moderator of the relationship between internet game addiction symptoms and attentional disengagement bias. Further analyses revealed a positive relationship between internet game addiction symptoms and attentional disengagement bias only among those with low levels of AC-shifting ability. Contrary to our expectations, AC-shifting also moderated the relationship between internet gaming disorder and attentional engagement bias. The positive relationship between internet game addiction symptoms and attentional engagement bias only appeared among those with low levels of AC-shifting ability. Individual differences in AC-focusing did not moderate the relationship between internet gaming disorder and any attentional bias.

**Conclusion:**

This study confirmed that the greater the symptoms of game addiction, the stronger the attentional bias, especially in individuals with low AC-shifting ability. Therefore, it is necessary to examine sub-factors of AC in understanding the nature of attentional bias mechanisms in the development of internet game addiction and consider it as a psychological intervention to improve attentional bias.

## Introduction

The tendency to preferentially attend to game information is hypothesized to play an important role in the development of internet game addiction tendency (1). This tendency, namely, attentional bias, towards addiction-related cues is a significant mechanism that increases cravings and leads to engaging in addictive behaviors (2). Intervention targeting attentional bias is crucial in the prevention of addictive behaviors, in that individuals are less likely to engage with stimuli to which they do not attend (3). However, there is a lack of research exploring the sub-processes of attentional bias and the factors influencing them. The extant studies show mixed results in both the nature and magnitude of attentional biases that characterize individuals with elevated internet game addiction tendency (4–6). Because attentional processes are important in understanding the development and maintenance of internet game addiction, it would be informative to elucidate variables that account for differential attentional patterns to game cues. Therefore, the purpose of the present study was to examine whether differences in subcomponents of attentional control, the ability to use attentional resources in processing emotional stimuli, account in part for the sub-process of attentional bias observed in individuals with internet game addiction tendency.

Research based on the Stroop task has identified that individuals with tendencies toward internet gaming addiction exhibit significantly longer reaction times to game-related words compared to neutral words (4). Similarly, dot-probe studies have provided evidence of attentional bias towards game-related stimuli, demonstrating that individuals with pathological internet gaming addiction show faster attentional engagement with game-related images, with activation in specific brain regions contributing to this attentional bias (7). Among the studies adopting classical paradigms such as the Stroop or dot-probe task, some studies have found evidence of attention bias toward stimuli directly related to the game, while others have not (8, 9). The mixed research results may stem from limitations of the tasks that failed to dissect attentional bias into sub-processes and oversight of individual factors contributing to diverse patterns of attentional bias. In this study, to accurately and simultaneously measure attentional engagement and disengagement, the attentional response to distal versus proximal emotional information (ARDPEI, 10) was employed. ARDPEI allows for the assessment of attentional engagement and disengagement by positioning attention either close to or distant from the stimulus of interest, followed by the presentation of a probe stimulus. Although the salience of internet game-related stimuli may differ from that of neutral stimuli, the literature frequently compares the attentional patterns associated with addiction-related stimuli and neutral stimuli (5, 10–14). Accordingly, this study aimed to investigate how individuals with tendencies toward internet game addiction exhibit differences in attentional engagement and disengagement with respect to internet game-related stimuli.

Posner and DiGirolamo (15) disentangled two different biases and define one of them as attentional engagement bias, which means a facilitated attentional shift toward addiction-related stimuli, and the other as attentional disengagement bias, which means difficulties shifting attention away from addiction-related stimuli. Posner argued that both attentional engagement and attentional disengagement bias are automatic processes, implying that both are induced by the emotional valence of stimuli automatically. However, subsequent research have suggested that attentional disengagement bias and engagement bias could reflect different levels of cognitive control (16–18). Some studies suggested that attentional disengagement bias, compared to attentional engagement, reflects higher-level cognitive processes (19–25). Attentional disengagement is often associated with the active inhibition or disengagement of attention from a distracting or irrelevant stimulus. This process is thought to be affected by top-down control mechanisms that regulate attention. On the other hand, attentional engagement bias is often associated with automatic or bottom-up processes that capture attention (11). While attentional engagement biases can also be influenced by top-down factors, they are generally considered to have a stronger bottom-up component (26). Overall, the current research suggests that attentional disengagement bias is more closely associated with top-down processes, while attentional engagement bias may involve a stronger bottom-up influence.

One of the top-down mechanisms, which is closely associated with attentional bias, is attentional control (AC; 27–32). AC refers to the ability to direct and regulate attention according to one’s goals and intentions. It involves the capacity to allocate attentional resources selectively, maintain focus, and resist distractions. There are two dimensions underlying attentional control, namely, shifting and focusing (33, 34). The shifting measures the ability to switch attention flexibly across different stimuli or tasks. It involves disengaging attention from one focus and redirecting it to another. The focusing dimension measures the ability to sustain attention on a particular stimulus or task over time. It involves inhibiting distractions and maintaining a consistent focus on the relevant information. Attentional control is a key aspect of executive functioning and plays a crucial role in various cognitive processes, including working memory, problem-solving, and decision-making. The lack of AC in substance or substance users makes it difficult for them to focus on negative outcomes that addiction cues can cause, suppress attention to addiction cues, and shift attention to stimuli other than addictive substances, resulting in difficulty resisting cravings and making healthy choices. Individuals with high attentional control are more likely to make long-term decisions that align with future goals, such as distancing themselves from addiction cues, while those with low attentional control are more likely to make impulsive and short-sighted decisions to fulfill immediate desires (35). In summary, the deficiency in AC impairs our ability to regulate attention towards rewarding stimuli, expectations, and motivations, and makes it difficult to engage in other hobbies and divert attention away from addiction cues.

Recent studies have suggested that AC has a moderating effect on attentional bias. Studies in substance use disorders have shown that goal-directed attentional control has been shown to modulate the effect of learned value on attention (26). Research in anxiety disorder has suggested that the moderating effect of attentional control on attentional bias could change depending on the sub-process of attentional bias. Derryberry and Reed (19) found that anxious participants with high attentional control are better at disengaging attention from emotionally arousing stimuli. Taylor and colleagues (23) revealed that at low levels of AC-shifting, individuals with high levels of social anxiety tended to have difficulties in disengaging their attention from threat-inducing social cues compared to neutral ones. In contrast, at high levels of AC-shifting, individuals with a high level of social anxiety disengaged their attention from threat-inducing social cues more quickly compared to neutral stimuli. However, another component of attentional control, AC-focusing, did not moderate the relationship between social anxiety and attentional bias. In conclusion, studies revealed that attentional disengagement could be moderated by AC-shifting, while attentional engagement was not. Both anxiety disorders and addiction involve cognitive processes that interact through a dual-process model (36–38), in which both automatic and voluntary systems play a role and both disorders are similar in that attentional bias to emotional stimuli is a major mechanism in development of disease. The dual-process model provides a significant theoretical framework across various fields such as learning, behavior modification, addiction, therapy, and education. According to the dual-process model, human behavior is influenced by the interaction between automatic processes and intentional processes. Automatic processes are associated with classical conditioning, wherein automatic associations between stimuli and responses are formed. These processes occur unconsciously and elicit automatic responses to learned stimuli. Intentional processes are related to operant conditioning and involve the ability to predict the consequences of actions and adjust behavior accordingly. These processes are conscious, and goal directed. Therefore, the moderating effect of attentional control on attentional bias, as observed in anxiety disorder research, is likely to apply to internet gaming addiction tendency as well.

Similarly, recent studies propose that the brain reward pathways involved in substance addiction and behavioral addictions, such as internet gaming addiction, share significant similarities (39). Excessive engagement in internet gaming can elicit natural responses in the brain’s reward system, where the dopamine system—known to be associated with reward—is closely linked to emotions, motivation, and goal-directed behavior. This tendency can be explained by the theory of incentive sensitization, which suggests that hypersensitivity to rewarding properties leads to an excessive focus on and pursuit of these stimuli. Rewarding stimuli can include substances or specific behaviors, both of which have the potential to induce addiction. When individuals are continuously exposed to such stimuli and experience pleasure, the brain forms associations between the stimuli and positive reinforcement. As reinforcement is repeated, these associations strengthen, leading individuals to develop a heightened motivation to seek and engage with the rewarding stimuli, resulting in conditioned responses (39, 40). Similarly, excessive involvement in internet gaming activates the brain’s dopamine system, providing individuals with excitement and pleasure. These rewarding experiences increase motivation and, consequently, the tendency to engage in or become absorbed by these activities.

Although AC has been shown to modulate attentional bias related to social anxiety (13), there are still many questions remaining unanswered in addiction. First, although studies have examined the influence of AC on the relationship between social anxiety and attentional bias, to our knowledge, there are no studies that have examined the influence of AC on the relationship between internet game addiction tendency and attentional bias. Addressing this issue may uncover the factor that has contributed to the variability in extant studies investigating the relationship between internet game addiction tendency and attentional biases. Second, exploring the subcomponents of both AC and attentional biases towards game-related cues could offer a more detailed comprehension of the underlying mechanisms involved in information processing within internet gaming disorder. This may lead to tailored interventions and treatments that take individual differences in attentional patterns into account.

The objective of this study was to examine whether subcomponents of attentional control (AC), specifically AC-shifting and AC-focusing, moderate the relationship between internet gaming disorder symptoms and attentional bias to game-relevant information. Based on AC theory (37) and previous findings (19, 23, 41), it was hypothesized that individual differences in AC-shifting and AC-focusing abilities would moderate the relationship between internet gaming disorder symptoms and attentional engagement or disengagement from game-related information. The specific hypotheses were as follows: (1) Among individuals with internet gaming disorder, those with lower AC-shifting ability would have greater difficulty disengaging from game cues. (2) AC-shifting would weakly moderate the relationship between internet gaming disorder symptoms and attentional engagement bias (42), as attentional engagement biases are assumed to reflect more bottom-up (stimulus-driven) processes compared to attentional disengagement. (3) The AC-focusing scale would not moderate the relationship between internet gaming disorder and measures of attentional engagement and disengagement. This prediction is based on previous anxiety research suggesting that the thematic content of the AC-focusing scale, which represents the ability to maintain attentional allocation on a target stimulus, does not have a moderating effect on attentional engagement and disengagement biases.

## Materials and methods

### Participants

The sample size was computed *a priori* with G*Power (version 3.1.9.2, Kiel, Germany). With *α* = 0.05, *f* = 0.15, and a power of 0.90, it yielded an overall sample size of 73 participants. Only male participants were included in the study to control the potential effect of gender on the relationship between attention to game-related cues and gaming behavior (43). To control for potential confounding variables, the types of internet game was unified by League of Legend (hereafter referred to as LOL). Participants were recruited through advertisements posted on university bulletin board and online forums in Korea. The advertisements described the study as targeting “individuals playing League of Legends (LOL)” and stated that a participation compensation of 15,000 KRW would be provided after 40–50 min of participation in the experiment. A total of 152 university students completed the initial screening questions. Among them, those who meet the following criteria were excluded: (1) students who play other games than LOL and play LOL less than 5 h a week (44); (2) students who have past diagnosis and treatment history of psychiatric/neurological disorders (e.g., depression, anxiety, schizophrenia, ADHD, and substance dependence); (3) students who have an intellectual problem or history of brain damage; (4) students who participate or have participated in gambling, are using or have used illegal drugs, or have other types of addiction; and (5) students who have vision problems. As a result, 75 participants were recruited.

### Questionnaires and measurement

#### Attentional control scale

The ACS (19) is a 20-item self-report questionnaire used to measure individual differences in attentional regulation and asks the individual to rate how they feel about situations related to concentrating and attentional flexibility on a four-point scale (1 = almost never to 4 = always). This questionnaire has shown to be a valid measure of attentional regulation (33, 34) and to have good internal consistency (*α* = 0.88; 19). Following prior factor analytic research (33), we used the two subscales of the ACS, attentional shifting (10 items) and attentional focusing (9 items). The Korean version translated by Yoon (45) was used. In the validity study, Cronbach’s *α* was 0.84, and in present study, Cronbach’s *α* was 0.92.

#### Internet addiction test

The IAT is created by the Center for On-Line Addiction by Young (1996). IAT consists of a total of 20 questions and measures obsessive use, behavioral problems, and emotional changes related to the internet or communication use. This scale is a five-point Likert scale (0 = not applicable to 5 = always), and the higher the score, the higher the level of internet game addiction. This study used the Korean version of IAT translated by Yoon (46). In the validity study (47), the Cronbach *α* coefficient of the first test was 0.93 and the second test showed a reliability of 0.91, In this study, Cronbach *α* was 0.91.

#### Attentional response to distal vs. proximal emotional information task

**Figure 1:**
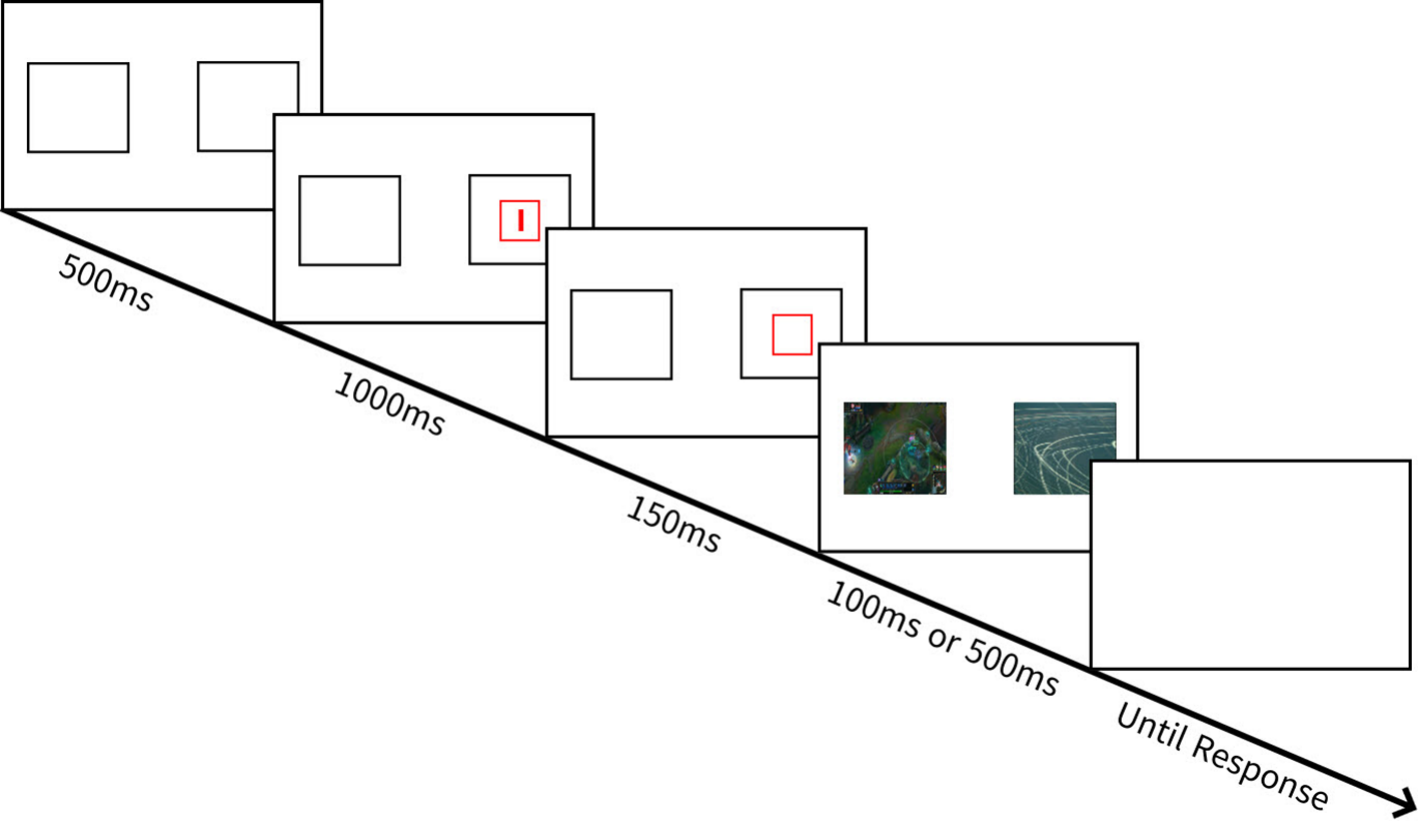
Example of ARDPEI task.

The ARDPEI task (10) was employed to assess biased attentional engagement with, and biased attentional disengagement from, gaming images relative to the daily scenery images (see **Figure 1**). Task specifications (i.e., number of trials, counterbalancing, and stimulus exposure duration) were consistent with Grafton and MacLeod (10), which is the initial research utilizing the ARDPEI task. Each trial commenced with the 1,000-ms presentation of two white rectangle outlines, each measuring 85 × 85 mm: one displayed 25 mm to the left and the other 25 mm to the right of screen center. A smaller red rectangular outline, measuring 20 × 20 mm, appeared in either of the bigger white rectangles with equal frequency. Participants were required to initially focus their attention within this red rectangle. One thousand milliseconds later, an anchor cue briefly appeared (150 ms) within this attended region. The anchor cue was a small red line (5 mm), which, with equal frequency, was either horizontal or vertical in orientation, and participants were required to note its orientation. Immediately after this anchor probe disappeared, an image pair consisting of a representational image (i.e., game or daily scenery) and non-representational image (i.e., abstract art) was presented for 100 ms on half of the trials, and on another half of the trials, it was exposed for 500 ms. On half of the trials, the representational image appeared in the opposite location to that where the participant’s initial attention had been anchored at the beginning of the trial, and these trials served to assess selective attentional engagement with distal images of game compared to daily scenery. On the other half of the trials, the representational image appeared in the same location where the participant’s initial attention had been anchored at the beginning of the trial, and these trials served to assess selective attentional disengagement from proximal images of game compared to daily scenery. Following the termination of the image display, a target probe appeared in either of the two screen locations, with equal frequency. The target probe was also a small (5 mm) red line, either horizontal or vertical in orientation. Participants were required to process, as quickly as possible, whether the orientation of the target probe matched that of the previously exposed anchor cue, which was the case on 50% of the trials. The participant responded using the mouse by pressing either the left or right mouse button to respectively indicate that the orientation of target probe did or did not match that of anchor probe. The latency to make this probe discrimination response and accuracy was recorded. Upon detection of this response, the screen was cleared for 1,000 ms, and then the next trial commenced. In total, there are two blocks of 128 trials, with each block separated by a self-timed rest period. Within each block, eight conditions (refer to **Table 1**) were delivered. The order of these conditions was counterbalanced across participants.

**Table 1:** Types of trial for each category image.

No.	Image position	Image time (ms)	Target image position
1	Distal	100	Different
2	Distal	100	Same
3	Distal	500	Different
4	Distal	500	Same
5	Proximal	100	Different
6	Proximal	100	Same
7	Proximal	500	Different
8	Proximal	500	Same

#### Experimental stimuli

The experimental design required 256 images, half of which were “representational” and half of which were “non-representational”. Of the 128 representational images, 64 were game images and the other 64 were neutral images. Screenshots of LOL were used as game stimuli. To validate the game stimuli, 15 people who play LOLs rated each of the 160 images individually on a five-point Likert scale (0 = not at all to 5 = extremely). The results of rating for the relevance, craving, arousal, and valence of pictures are given in **Table 2**. Landscape images obtained from Google Image Search were used as neutral stimuli. To ensure that attentional bias was not triggered by the color and salience of the stimuli, landscape images went through the process of adjusting color and salience similar to game stimuli. The non-representational images were drawn from initial studies that sought to validate the ARDPEI task (10). The studies obtained abstract images by searching the term “abstract art” on Google Image Search for open-source images. These images were rated in the way the International Affective Picture System (IAPS) images were rated. In the previous study, the mean of the emotional valence of the obtained abstract images was 4.77 (SD = 0.23).

**Table 2:** Relevance, craving, arousal, and valence of stimuli in the ARDPEI task.

	Relevance	Craving	Arousal	Positive valence	Negative valence
Game	4.86 (0.09)	3.27 (0.21)	2.87 (0.28)	3.08 (0.38)	1.87 (0.34)
Neutral	l.16 (0.12)	1.83 (0.23)	1.72 (0.19)	1.48 (0.17)	1.15 (0.12)

Mean (standard deviation). ARDPEI task, attentional response to distal vs. proximal emotional information task.

### Procedure

All participants were asked to restrain from playing games for 4 h before the experiment. Upon arrival at the laboratory, the participants were asked to complete the informed consent. After that, participants were seated approximately 60 cm in front of the computer screen and given instructions for the ARDPEI task. Participants completed a short practice comprising 32 trials that included only abstract images not used within the assessment version of the task. After confirming that the participants fully understand the task, participants completed the ARDPEI task. After completing the ARDPEI task, the self-report questionnaires including IAT and ACS were administered. The participants were then debriefed and received monetary compensation (15,000 KRW). The whole session including the ARDPEI task, completion of self-questionnaires, and debriefing lasted approximately 40 min in total. This study was approved by the Institutional Review Board of Chung Ang University (IRB No. 1041078-20230401-HR-093).

### Data analysis

All statistical analyses were analyzed using IBM SPSS Statistics for Windows version 26. Prior to the analyses, all data went through a screening and cleaning process to confirm missing data, occurrence of outliers, and volition of statistical assumptions.

For the data analysis of the ARDPEI, a participant who was found to be an outlier in terms of the probe discrimination accuracy, which fell more than 2.58 SD below the mean, was removed. Through this step, five participants were removed, and the remaining participants displayed reassuringly high rates of accuracy on this task, averaging less than 5% of errors, indicating that they complied with the requirement to initially attend to the locus of the anchor probe and to subsequently discriminate the identity of the target of the target probe. Before computing the engagement bias index and disengagement bias index from the ARDPEI task probe discrimination latencies, outlier probe discrimination latencies that fell more than 2.58 SD from any participant’s mean probe discrimination latency for that particular experimental condition were eliminated. This led to the exclusion of 2.34% of the latencies. Lastly, reaction times faster than 200 ms, which are most likely anticipations errors, were deleted. This was limited to five trials in the engagement bias condition and eight trials in the Disengagement condition. Through these steps, the engagement bias index and disengagement bias index were extracted and calculated. Hierarchical regression analyses were used to examine the hypothesis that different levels of AC would moderate the relationship between internet gaming disorder symptoms and attentional processing of game information. Because our study intend to examine the influence of sub-facets of AC on subcomponents of attentional bias to game-related cue, separate regression models were tested for attentional engagement and disengagement bias index as well as AC-shifting and AC-focusing subscales. In each model, the level of internet game addiction tendency (IAT score) and AC subscale scores (shifting or focusing) was used as predictors.

Attentional engagement and disengagement index was used as the dependent variables: an engagement bias for the short (100 ms) and long (500 ms) category image time trials as well as a disengagement bias for the short and long image time trials. The engagement bias indicates how quickly participants can respond when game images are presented compared to when neutral images are presented. To determine this, we calculated the difference in reaction times when participants responded to probes located in different positions from the game images as the baseline, and compared the reaction times when game images were presented to when neutral images were presented. The disengagement bias index was computed from those trials in which the representational image appeared in the same locus as that on which the anchor cue had the participants’ initial attention focused. Specifically, this index reflected the difference in reaction times of trials where the probe appears in the same position as the image and the trials where the probe was in the opposite position.

Prior to the analyses, continuous predictor variables included in interaction terms were centered (48). The two predictor variables, IAT and AC, were entered separately in steps 1 and 2 of the regression equation. The IAT × AC interaction term was entered in step 3 of the regression analysis. A region of significance analysis using the Johnson–Neyman technique (49) was conducted to probe the significant interactions. This approach provides the specific values of the moderator variable at which the relationship between internet game addiction tendency and attentional bias to game information goes from non-significant to significant at *α* = 0.05. The Johnson–Neyman procedure was implemented using an SPSS macro.

## Results

### Demographic and clinical characteristics

The demographic characteristics and status of game usage of the participants were analyzed. The participants of this study were all male LOL game users, with an average age of 21.89 years (SD = 2.54). The average game usage period of respondents was 146.43 months (SD = 88). The average game usage time during the week was 8 h (SD = 6.12). Additionally, the mean response values for the IAT among the participants was 38.90 (SD = 19), while the mean scores for AC-shifting and AC-focusing were 17.90 (SD = 4.37) and 26.25 (SD = 3.43), respectively.

### Hierarchical regression analysis

In this section, the results of the hierarchical regression analyses performed are presented. These models considered IAT score as an independent variable, attentional engagement bias index or attentional disengagement bias index as a dependent variable, and AC score as a continuous moderator variable. Given the specificity of the hypotheses regarding the influence of sub-factors of AC on AEB, the sub-factors of AC, attentional shifting and attentional focusing, were tested separately as moderators.

#### AC-shifting as a moderator of the relationship between internet addiction test score and attentional engagement bias

**Figure 2:**
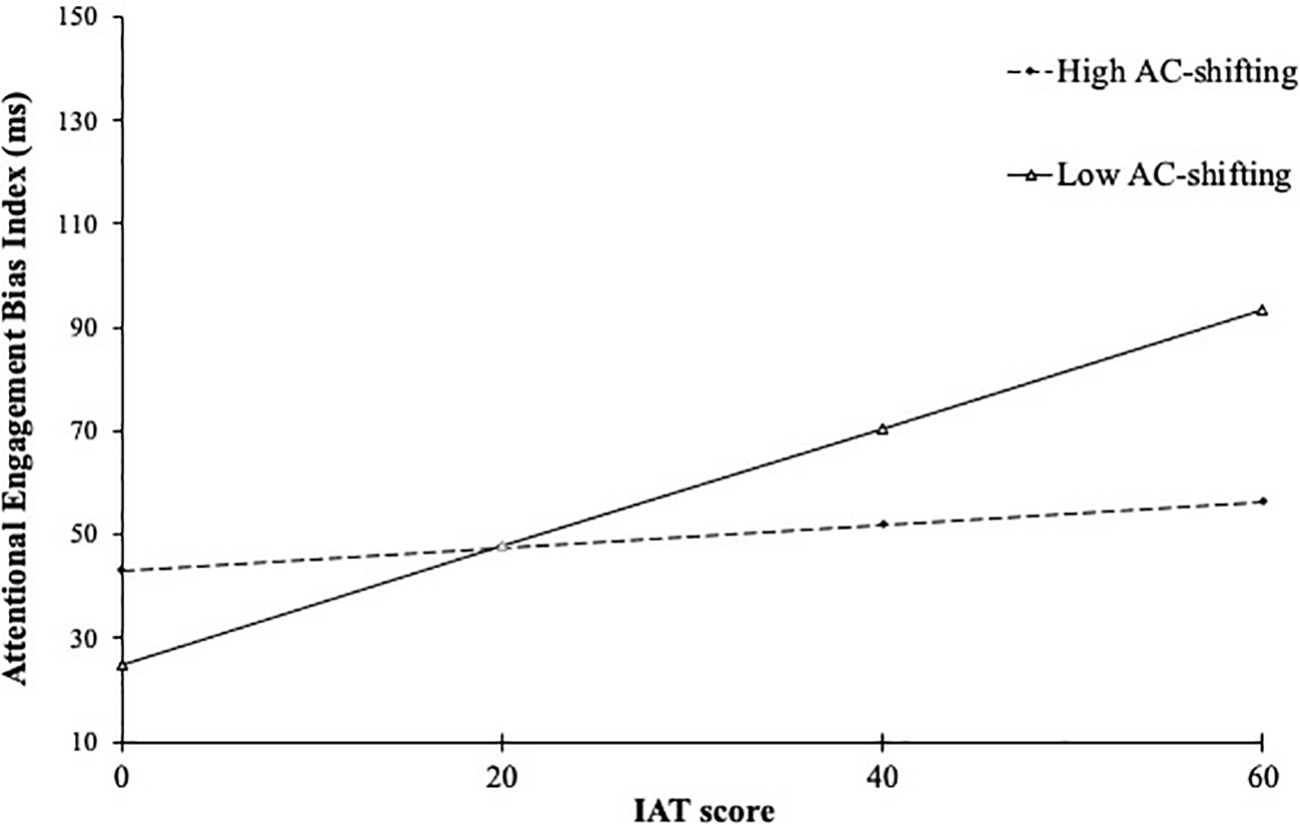
Simple slope of AC-shifting score moderation effect on internet addiction test score and attentional engagement bias index. IAT, Internet Addiction Test; AC-shifting, score of shifting factors in the Attentional Control Scale; High AC-shifting, AC-shifting score higher than +1 SD, Low AC-shifting, AC-shifting score lower than –1 SD.

A moderation analysis was conducted to test if the attentional shifting moderates the relationship between internet gaming disorder symptoms and attentional engagement bias to game stimuli. **Table 3** shows the results of the analysis that consider IAT score as an independent variable, attentional engagement bias index as a dependent variable, and the AC-shifting score as a continuous moderator variable. The moderation effect of AC-shifting was significant in contradiction to our hypothesis, examining the relationship between individual differences in AC-shifting and engagement from game information (Δ $R^{2}$ = 0.06, *p* = 0.04). This result indicates that individual differences in AC-shifting moderated the relationship between the level of IAT and attentional engagement bias to game stimuli (see **Figure 2**). A region of significance analysis identified 19.40 on the AC-shifting measure as points of transition going from a statistically non-significant to significant relationship between IAT and attentional engagement index. Specifically, this analysis revealed that for AC-shifting scores less than 19.40 to the lowest value observed (13.55), level of internet gaming disorder was positively associated with attentional engagement index. That is, higher levels of internet gaming disorder symptoms were associated with significantly greater attentional engagement bias scores for game cues. On the other hand, for AC-shifting scores greater than 19.40 to the largest observed value (22.55), level of internet gaming disorder was not associated with attentional engagement index.

**Table 3:** Hierarchical regression analyses of individual differences in internet gaming disorder and AC-shifting predicting attentional engagement bias scores for game stimuli.

	Step 1		Step 2		Step 3	
	*B* (SE)	*β*	*B* (SE)	*β*	*B* (SE)	*β*
IAT	8.04 (1.94)	0.51	7.75 (1.95)	0.49	7.87 (1.88)	0.50
AC-shifting			5.82 (5.19)	0.14	−0.35 (6.62)	−0.08
IAT × AC-shifting					−1.96 (0.91)	−0.33
Δ $R^{2}$	0.26***		0.02		0.06*	

AC, Attentional Control Scale; IAT, Internet Addiction Test.

*p < 0.05, ***p < 0.001.

#### AC-focusing as a moderator of the relationship between internet addiction test score and attentional engagement bias

**Figure 3:**
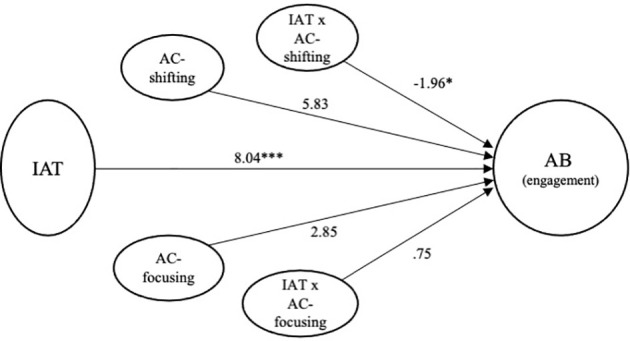
Final model for internet addiction test score and attentional control predicting attentional engagement bias. AC, Attentional Control Scale; IAT, Internet Addiction Test; AB, Attentional Bias. *p < 0.05, ***p < 0.001.

A moderation analysis was conducted to test if the attentional focusing moderates the relationship between internet gaming disorder symptoms and attentional engagement bias to game stimuli (refer to **Table 4**). The regression analysis examining AC-focusing as a moderator of the relationship between internet gaming disorder and attentional engagement index revealed that the IAT × AC-focusing interaction was not significant (Δ $R^{2}$ = 0.01, *p* = 0.35). These findings indicated that individual differences in AC-focusing abilities did not influence the relationship between internet gaming disorder symptoms and attentional engagement bias index (see **Figure 3**).

**Table 4:** Hierarchical regression analyses of individual differences in internet gaming disorder and AC-focusing predicting attentional engagement bias scores for game stimuli.

	Step 1		Step 2		Step 3	
	*B* (SE)	*β*	*B* (SE)	*β*	*B* (SE)	*β*
IAT	8.04 (1.94)	0.51	7.76 (1.98)	0.49	7.74 (1.98)	0.49
AC-focusing			2.85 (3.59)	0.10	6.17 (5.00)	0.21
IAT × AC-focusing					0.75 (0.78)	0.16
Δ $R^{2}$	0.26***		0.01		0.01	

AC, Attentional Control Scale; IAT, Internet Addiction Test.

***p < 0.001.

#### AC-shifting as a moderator of the relationship between internet addiction test score and attentional disengagement bias

**Figure 4:**
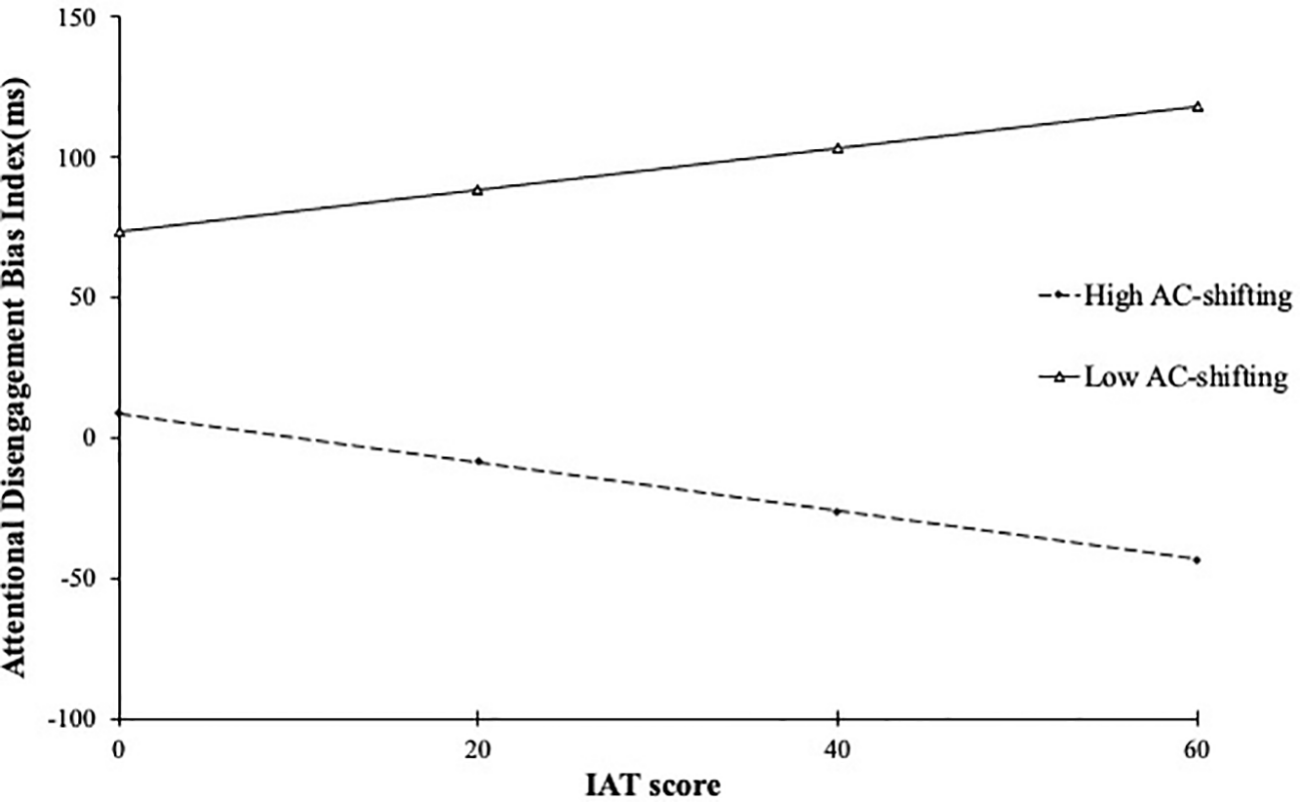
Simple slope of attentional control score moderation effect on internet addiction test score and attentional disengagement bias index. IAT, Internet Addiction Test; AC-shifting, score of shifting factors in the Attentional Control Scale; High AC-shifting, AC-shifting score higher than +1 SD; Low AC-shifting, AC-shifting score lower than –1 SD.

A moderation analysis was conducted to test if the attentional shifting moderates the relationship between internet gaming disorder symptoms and attentional disengagement bias to game stimuli. IAT score was used as an independent variable, disengagement bias index was used as a dependent variable, and the AC-shifting score was used as a continuous moderator variable (refer to **Table 5**). The moderation effect of AC-shifting was significant in line with the hypothesis examining the relationship between individual differences in AC-shifting abilities and disengagement from game information. Results of this analysis revealed a significant IAT × AC-shifting interaction (Δ $R^{2}$ = 0.21, *p* = 0.006), which indicated that individual differences in AC-shifting abilities moderated the relationship between level of internet gaming disorder and attentional disengagement bias for negative social stimuli (see **Figure 4**). A region of significance analysis identified that for AC-shifting scores less than 16.41 to the lowest value observed (12.73), level of internet gaming disorder was positively associated with attentional disengagement index. That is, higher levels of internet gaming disorder symptoms were associated with significantly greater attentional disengagement bias scores for game cues. For AC-shifting scores greater than 16.41, the relationship between internet gaming disorder symptoms and attentional disengagement bias scores was not significant.

**Table 5:** Hierarchical regression analyses of individual differences in internet gaming disorder and AC-shifting predicting attentional disengagement bias scores for game stimuli.

	Step 1		Step 2		Step 3	
	*B* (SE)	*β*	*B* (SE)	*β*	*B* (SE)	*β*
IAT	3.12 (2.01)	0.20	2.71 (1.91)	0.17	1.46 (1.86)	0.92
AC-shifting			−12.27 (4.51)	−0.33	−11.84 (4.26)	−0.32
IAT × AC-shifting					−2.18 (0.76)	−0.34
Δ $R^{2}$	0.04		0.11**		0.11**	

AC, Attentional Control Scale; IAT, Internet Addiction Test.

**p < 0.01.

#### AC-focusing as a moderator of the relationship between internet addiction test score and attentional disengagement bias

**Figure 5:**
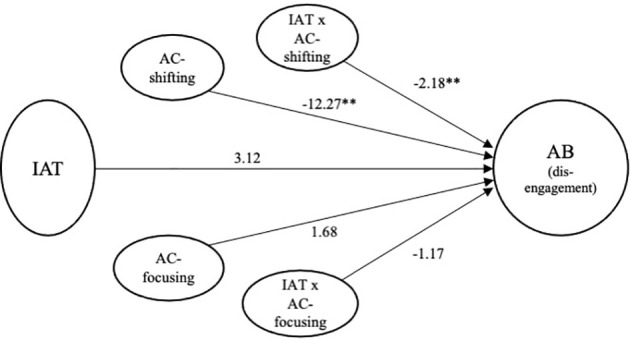
Final model for internet addiction test score and attentional control predicting attentional disengagement bias. AC, Attentional Control Scale; IAT, Internet Addiction Test; AB, Attentional Bias. **p < 0.01.

A moderation analysis was conducted to test if the attentional focusing moderates the relationship between internet gaming disorder symptoms and attentional disengagement bias to game stimuli (refer to **Table 6**). IAT score was used as an independent variable, disengagement bias index was used as a dependent variable, and the AC-focusing score was used as a continuous moderator variable. The regression analysis revealed that the IAT × AC-focusing interaction was not significant (Δ $R^{2}$ = 0.04, *p* = 0.14). This finding indicated that individual differences in AC-focusing abilities did not influence the relationship between internet gaming disorder symptoms and attentional disengagement scores (see **Figure 5**).

**Table 6:** Hierarchical regression analyses of individual differences in internet gaming disorder and AC-focusing predicting attentional disengagement bias scores for game stimuli.

	Step 1		Step 2		Step 3	
	*B* (SE)	*β*	*B* (SE)	*β*	*B* (SE)	*β*
IAT	3.12 (2.01)	0.20	3.00 (2.04)	0.19	2.89 (2.02)	0.18
AC-focusing			1.68 (3.77)	0.06	0.44 (3.82)	0.02
IAT × AC-focusing					−1.17 (0.79)	-0.19
Δ $R^{2}$	0.04		0.003		0.04	

AC, Attentional Control Scale; IAT, Internet Addiction Test.

## Discussion

The goal of the current study was to examine whether individual differences in subcomponents of AC moderated the relationship between the level of internet gaming disorder symptoms and attentional engagement and disengagement bias to game cues. The main finding was that individual variability in the ability to shift attentional allocation moderated the association between internet gaming disorder and attentional engagement as well as the association between internet gaming disorder and disengagement to game cues. This study adds to a growing literature underscoring the importance of AC processes in understanding the nature of attentional mechanisms (13, 19, 50–52). The current findings extend the extant literature by showing the importance of investigating subcomponents of both AC and attentional bias mechanisms in addiction.

Individual differences in one dimension of AC, AC-shifting, were associated with differential patterns of attentional disengagement from game cues at high levels of internet gaming disorder, but individual differences in the other dimension, AC-focusing, were not. The regions of significance analysis revealed that at low levels of AC-shifting, higher levels of internet gaming disorder were associated with greater difficulties disengaging attention from game cues. These findings converge with recent studies demonstrating a link between internet gaming addiction and attentional disengagement biases to addiction-related information (6). In contrast, from moderate to high levels of AC-shifting, increasing levels of internet gaming disorder were not associated with attentional disengagement from game cues. These findings converge with a study of cocaine-dependent subjects, suggesting that the attentional bias was associated with inhibitory control in cocaine-dependent subjects but not in control subjects (12). Taken together, this pattern of findings mirrors those reported in recent studies (12, 18, 53, 54) in which general self-control moderated the relationship between attentional biases to rewarding stimulus and internet gaming addiction. In other words, individuals with weaker inhibitory control may exhibit heightened attentional bias, which may contribute to the maintenance or escalation of addictive behaviors.

In contrast to predictions, AC-shifting moderates the relationship between internet game addiction tendency and attentional engagement bias. Studies have suggested that the gaming experience could influence visual processing and certain visual attentional abilities (55–57). For example, Boot and his colleagues (56) investigated visual search skills and reaction times to visual stimuli, and the ability to process rapid visual information of game players was higher compared to non-gamers. Therefore, in subsequent studies, appropriate stimulus presentation times should be adopted to measure attention engagement in consideration of the attention characteristics of game users.

The effect of poor AC-shifting on difficulties disengaging attention from game stimuli in individuals with high levels of internet gaming disorder could be explained by the dual process model. According to dual process models of cognitive processes (37, 42), addictive behaviors are influenced by the interaction between two distinct cognitive processes: the impulsive or automatic system and the reflective or controlled system. The game information is salient for individuals with heightened internet gaming disorder; such information is likely to activate impulsive systems. Thus, game information preferentially demands attentional resources. However, individuals with moderate to heightened AC could interrupt those reactions of the impulsive process so that they show weaker disengagement bias (23).

Future research has the potential to expand upon the current study in various ways. First, it is crucial to acknowledge that the present sample consisted of male undergraduate students, necessitating the examination of generalizability to the other age range, sex, and clinical samples. Second, although AC was assessed using a validated scale, it relied on the participant’s subjective report. Moreover, consistent with prior studies (33), the internal consistency of the AC-shifting subscale was low, which suggests that all of the items may not be measuring the same latent variable, in this case, attentional shifting. Thus, it is important for future research to incorporate more objective measures when assessing AC. Third, this experiment was conducted adopting behavioral measurements, the ARDPEI task. Further research would measure the attentional bias mechanism by the psychophysiological indicator, such as an eye tracking device. Behavioral measurements do not directly measure eye movements or gaze patterns, so they have limitations in terms of the ability to provide detailed and precise measurements of attentional processes compared to eye trackers. This will allow for a more accurate assessment of attentional allocation and gaze patterns, providing detailed insights into visual attention. Finally, given that the current study design was cross-sectional, it is essential to note that causal inferences cannot be made and this study provides limited information on temporal changes. Nonetheless, this study provides valuable insights into the moderating role of AC on attentional bias associated with triggering cravings. However, to establish stronger causal relationships and overcome these limitations, longitudinal or experimental study designs may be more appropriate.

Given the confirmed importance of AC-shifting in this study, incorporating strategies to enhance attentional shifting abilities into game addiction treatment or prevention programs may be beneficial. Specifically, providing attentional shifting training to individuals with severe game addiction symptoms could help reduce attentional biases towards game stimuli. Furthermore, considering that the relationship between AC-shifting and internet gaming disorder may vary among individuals, intervention strategies should be tailored to the individual’s AC characteristics. For instance, individuals with lower AC-shifting may require more intensive attentional shifting training to mitigate attentional dispersion and regulate their responses to game stimuli.

## Conclusion

Overall, the current findings indicate that individual differences in AC may play a crucial role in comprehending the nature and intensity of attentional biases among individuals exhibiting symptoms of elevated internet gaming disorder. Furthermore, these differences may partly elucidate the inconsistent results observed in previous studies regarding the association between internet gaming disorder and attentional biases toward game-related cues. Furthermore, these findings underscore the need for additional investigation of sub-facets of AC and biases to addiction-related cues in addiction. Previous research has commonly treated AC as a unified construct; nevertheless, the recent findings indicate that distinct sub-facets of AC, particularly AC-shifting, play a crucial role in explaining the relationship between internet gaming disorder and attentional disengagement biases as well as between internet gaming disorder and attentional engagement biases. The AC-focusing subscale could not explain the pattern of attentional engagement or disengagement biases. Tasks that require sustained attentional focus [e.g., continuous performance task; (58)] may be more sensitive to revealing links between internet gaming disorder, AC-focusing, and attentional processes (59, 60). Future research is needed to address that issue.

## Data Availability

The original contributions presented in the study are included in the article/supplementary material. Further inquiries can be directed to the corresponding author.
